# Effective treatment of ductal carcinoma in situ with a HER-2-targeted alpha-particle emitting radionuclide in a preclinical model of human breast cancer

**DOI:** 10.18632/oncotarget.8949

**Published:** 2016-04-23

**Authors:** Takahiro Yoshida, Kideok Jin, Hong Song, Sunju Park, David L. Huso, Zhe Zhang, Han Liangfeng, Charles Zhu, Frank Bruchertseifer, Alfred Morgenstern, George Sgouros, Saraswati Sukumar

**Affiliations:** ^1^ Department of Oncology, Johns Hopkins University School of Medicine, Maryland, USA; ^2^ Department of Radiology and Radiological Science, Johns Hopkins University School of Medicine, Maryland, USA; ^3^ Department of Molecular and Comparative Pathobiology, Johns Hopkins University School of Medicine, Maryland, USA; ^4^ Department of Biomedical Engineering, Rutgers University, Piscataway, NJ, USA; ^5^ European Commission, Joint Research Centre, Institute for Transuranium Elements, Karlsruhe, Germany; ^6^ Current address: Department of Surgery, Tokushima University, 3-18-15 Kuramoto-cho, Tokushima, Japan

**Keywords:** intraductal, radioimmunotherapy, trastuzumab, ductal carcinoma in situ, breast cancer

## Abstract

The standard treatment for ductal carcinoma in situ (DCIS) of the breast is surgical resection, followed by radiation. Here, we tested localized therapy of DCIS in mice using the immunoconjugate ^225^Ac linked-trastuzumab delivered through the intraductal (i.duc) route. Trastuzumab targets HER-2/neu, while the alpha-emitter ^225^Ac (half-life, 10 days) delivers highly cytotoxic, focused doses of radiation to tumors. Systemic ^225^Ac, however, elicits hematologic toxicity and at high doses free ^213^Bi, generated by its decay, causes renal toxicity. I.duc delivery of the radioimmunoconjugate could bypass its systemic toxicity. Bioluminescent imaging showed that the therapeutic efficacy of intraductal ^225^Ac-trastuzumab (10-40 nCi per mammary gland; 30-120 nCi per mouse) in a DCIS model of human SUM225 cancer cells in NSG mice was significantly higher (p<0.0003) than intravenous (120 nCi per mouse) administration, with no kidney toxicity or loss of body weight. Our findings suggest that i.duc radioimmunotherapy using ^225^Ac-trastuzumab deserves greater attention for future clinical development as a treatment modality for early breast cancer.

## INTRODUCTION

Curing breast cancer is probably best achieved by eliminating preneoplasia and initiated cells in the breast ductal systems. Recently, the detection of DCIS has risen dramatically with the widespread use of screening mammography [1, 2]. The local recurrence rate after breast conservation therapy for DCIS is 8-14% [1, 2] and not negligible. HER-2, a transmembrane tyrosine kinase, is over-expressed in 30-40% of DCIS [3–5]. High HER-2 and/or Ki-67 expression are risk factors of local recurrence of DCIS [6].

Since targeted antibodies have successfully demonstrated activity in treating HER-2 positive breast cancer, conjugated antibodies have been explored for enhancing their potency [7, 8]. Our prior work has shown that radioimmunotherapy of mouse mammary tumor metastasis using α-emitter-labeled trastuzumab is superior to unlabeled-trastuzumab in terms of long-term survival [9, 10]. The efficacy of ^225^Ac, the parent of ^213^Bi, was significantly superior to the other treatments using common β-emitter particles [10]. However, antibody-bound ^225^Ac led to hematologic toxicity and free ^213^Bi arising from the decay of ^225^Ac in circulation accumulated in the kidneys and led to renal toxicity at high administered activities [9, 11]. We hypothesized that to circumvent renal and other systemic toxicity, localized delivery of ^225^Ac is an option worthy of investigation.

We have previously demonstrated the efficacy of intraductal administration (i.duc) of anticancer agents in the prevention and treatment of mammary tumors using rodent models [12–14]. The feasibility and safety of this method was demonstrated by us in Stage I breast cancer patients prior to mastectomy [14]. Since alpha particles can efficiently kill single cells and micro-tumors while sparing the surrounding normal tissues, delivery by the i.duc route could result in focusing its cytotoxic effects on the transformed, HER2 expressing ductal cells while also reducing systemic exposure [13, 14].

In this paper, we report the results of our investigation on whether the advantages of intraductal delivery could be leveraged for use in HER-2-targeted radioimmunotherapy for the treatment of mammary tumors in human HER-2 positive xenograft models. Based on promising results from preclinical studies, we propose that radioimmunotherapy by alpha-emitter radionuclides merits further investigation for future clinical applications.

## RESULTS

### Biodistribution of trastuzumab

**Figure 1:**
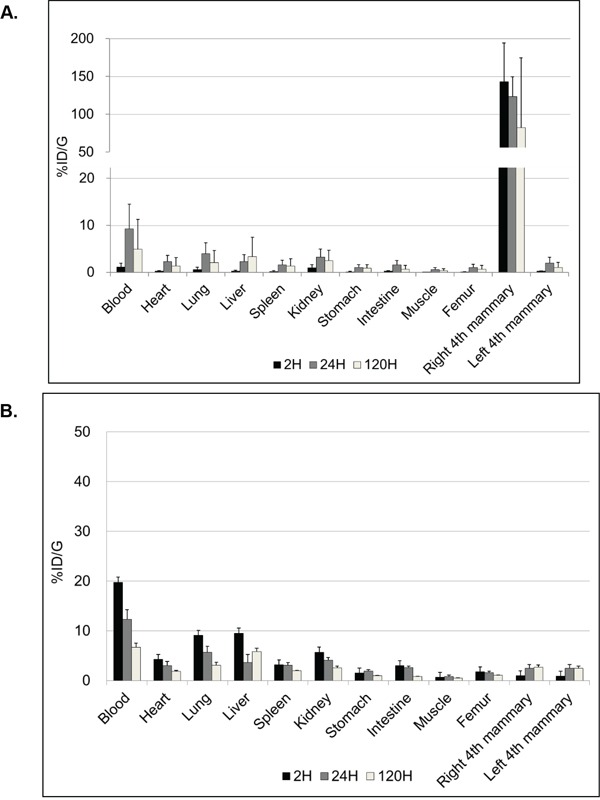
Biodistribution of trastuzumab %ID/g graphs show different patterns of biodistribution of trastuzumab at 2, 24, 120 hours after i.duc **A.** or tail vein **B.** injection (three mice per time point). The peak of %ID/g in the blood was 9.3 ± 5.3% at 24 hours after i.duc. %ID/g = (activity in each organ) X100/(actual injected activity) per gram for each organ. Data are expressed as %ID/g+/−SD.

The biodistribution of radio-immuno conjugates when injected locally by i.duc or systemically by i.v could be vastly different. Since this was the first attempt to administer the immunoconjugate i.duc, we studied biodistribution in more detail. We used ^111^In-trastuzumab to compare i.duc versus i.v biodistribution of the DOTA-conjugated antibody in female FVB/N mice, the parental strain of origin of the NSG mice. 5.0-20.0 μCi was injected into the tail vein (120 μl) or the right 4th mammary gland (40 μl). The mean percentage of i.duc-injected ^111^In-trastuzumab retained after administration in the right 4th mammary gland (%ID) was: 50.0% (SD, ± 25.0%) at 2 h, 44.5% (SD, ± 25.9%) at 24 h and 30.9% (SD, ± 33.4%) at 120 h, and %ID/G was subsequently high only in the i.duc-injected mammary gland (Figure 1A). In contrast, i.v administration resulted in peaking of drug levels in the blood within 2 h, and distribution to all the organs of the body in near equal concentrations (Figure 1B). The AUC in blood following i.duc administration was 620 ± 378 (SD) %ID/g-h versus 1220 ± 178 (SD) %ID/g-h for the i.v administration (p = 0.06). No acute hematological toxicity has been observed in prior murine studies with the blood AUC level observed following i.duc administration reported herein [11]. Thus, i.duc administration of the radioimmunoconjugate resulted in high localized concentration in the ductal system and reduced %ID/g in the other organs compared to systemic administration. These data suggested intraductal radioimmunotherapy may cause less systemic toxicity than the same dose of drug injected by the i.v. route. Further, a more sustained drug effect over a longer period of time may be achieved by the i.duc route.

### Inhibition of colony formation by radioimmunoconjugate

**Figure 2:**
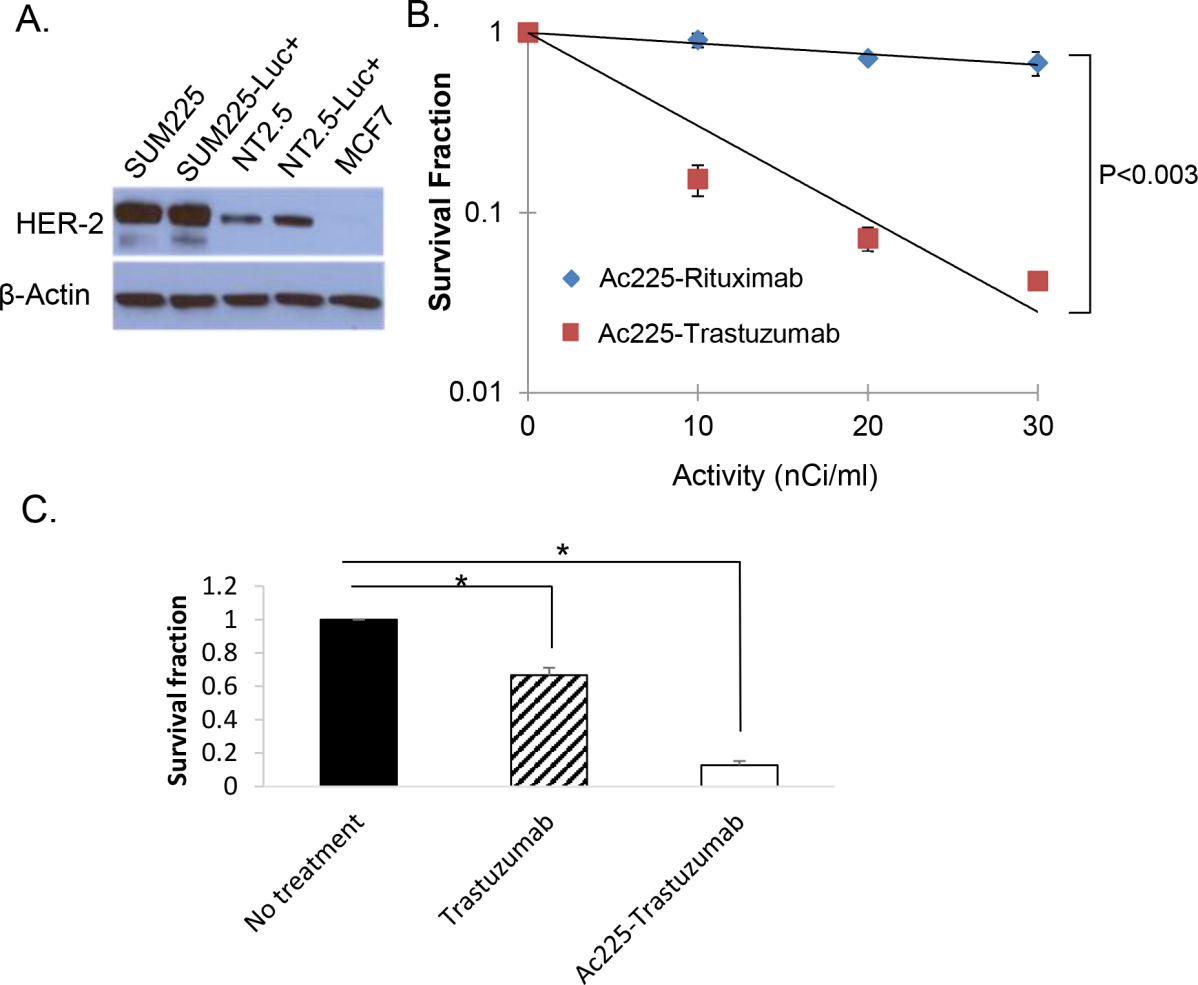
Colony formation assay with ^225^Ac-trastuzumab and ^225^Ac-rituximab **A.** Western blot analysis of HER-2 protein in HER2-overexpressing human breast cancer cell line, SUM225, rat Her2-positive transgenic mouse mammary tumor cell lines (NT2.5 and NT2.5-Luc+), and a low HER2-expressing human breast cancer cell line, MCF-7. **B.** Colony formation assay of SUM225-Luc+ cells with^225^Ac-trastuzumab and ^225^Ac-rituximab demonstrates HER-2-specific cell kill. Data is expressed as cell survival fraction ± SD. **C.** The contribution of unconjugated trastuzumab to cell death was tested by colony formation assay. SUM225-Luc+ cells were treated with cold trastuzumab (1 ug/ml) and 10 nCi/ml ^225^Ac-trastuzumab (1 ug/ml) for 1 hour. Data is expressed as cell survival fraction ± SD. (*P<0.001).

In order to investigate if ^225^Ac-trastuzumab can inhibit growth of SUM225-Luc+, we performed colony formation assays. To ascertain the level of expression of HER2 in the cell lines under study, Western blotting was performed using SUM225, MCF-7 breast cancer cells, and a cell line derived from a MMTV-rat-neu transgenic mouse mammary tumor (Figure 2A). The results of the colony formation assay using Her2-overexpressing SUM225-Luc+ cells showed that ^225^Ac-trastuzumab (D37 = 8.4 nCi/ml) was about 9 times more lethal in colony formation assays (Survival fraction of 10 nCi/ml ^225^Ac-trastuzumab = 0.91) compared to the non-specific monoclonal antibody ^225^Ac-rituximab (D37 = 71.4 nCi/ml) (Survival fraction of 10 nCi/ml ^225^Ac-Rituximab = 0.15) (Figure 2B). The contribution of unconjugated trastuzumab to cell death observed in this assay with the radioimmunoconjugate was evaluated by incubating SUM225-Luc+ cells with free trastuzumab (1 μg/ml) for 1 hour; colonies were observed in the range of 65-70% compared to 10% colonies observed with the radioimmunoconjugate (Figure 2C). The data showed that the specific affinity of trastuzumab to HER-2 was necessary to kill the HER-2 positive cells. Further, with the additive effect of ^225^Ac, the radioimmunoconjugate was superior to treatment with excess trastuzumab.

### Characterization of a mouse DCIS xenograft model, and intraductal immunotherapy

**Figure 3:**
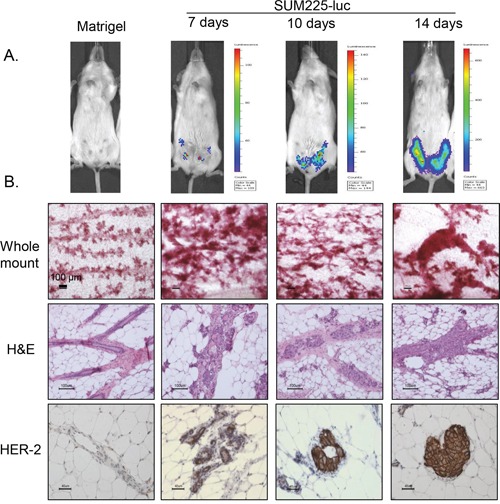
Tumor development in the SUM225-Luc+ DCIS xenograft model **A.** IVIS spectrum imaging shows representative tumor growth patterns in the mammary gland at 3 different time points after injection of SUM225-Luc+ cells through the teat. **B.** Panel 1: Whole mount staining performed 2 weeks after tumor cell inoculation showed enlarged buds and dilated ducts which increase in size with time. Panel 2: A single layer of SUM225 cells is seen attached to mouse epithelial layer of the mammary duct 1 week later, and tumor cells fill the ductal lumen 2 weeks later, as seen by H and E staining. Panel 3: Ductal outgrowths are HER2-positive by IHC. Bars indicate 100 μm for whole mount and H&E staining, and 40 μm for HER-2 staining.

We adopted the previously described mouse xenograft model for DCIS [17] with some modifications. SUM225-Luc+ breast cancer cells were injected i.duc into 4 inguinal mammary glands, and 10 μl of vehicle (Matrigel and complete media mixture) without cells was injected into the right and left 3rd mammary glands as controls. Nine NSG mice received intraductal injections; 3 mice each were examined by IVIS imaging (Figure S1) and were sacrificed at 7, 10 and 14 days after transplantation. IVIS imaging of the whole mouse detected tumor outgrowth in all four mammary glands by 7 days. At this time the outgrowths were not detected by palpation (Figure 3A). At each time point following sacrifice, in each mouse, 3 left mammary glands were examined by whole mount staining with carmine (Figure 3B, panel 1), the three right mammary glands were examined by hematoxylin and eosin (H and E) staining (Figure 3B, panel 2) and by immunohistochemistry for the HER2 antigen (Figure 3B, panel 3). With 3 mice for each time point, the distribution of tissues for the various tests was as follows: 3 matrigel controls and 6 tumor cell injected mammary glands for whole mount, 3 controls and 6 tests each for H and E staining and HER2 IHC accounting for a total of 27 measurements at each time point. One week after transplantation, growth of the intraductal xenografts was observed as progressive thickening of the ducts over time by the whole mount analysis and as one to two cell layers within the duct by H and E staining. By IHC, SUM225 cells stained strongly for HER2, and in some areas the i.duc implanted cells filled the duct. No microinvasion of the stroma by the tumor cells was observed. We concluded that the i.duc xenografts of SUM225-Luc+ cells in mice, one week after transplantation, served optimally as a model for DCIS for therapeutic studies.

**Figure 4:**
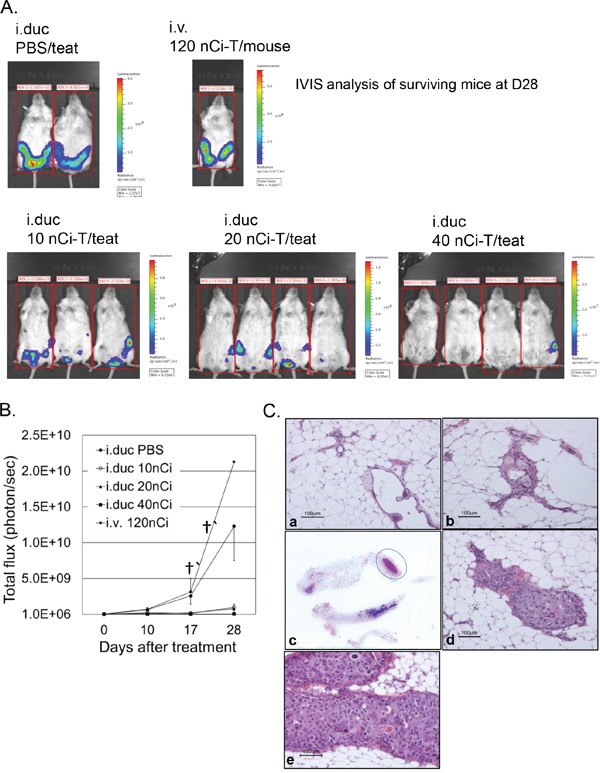
Effects of radioimmunotherapy on intraductal DCIS xenograft model **A.** IVIS spectrum imaging based evaluation of the mammary glands in the intact mice was performed just before treatment, and 10, 17 and 28 days after treatment with the immunoconjugate. IVIS shows all the mice in the 5 different groups at 28 days after treatment. Tumor growth is represented as total flux per mouse from IVIS at each time point. **B.** Data are expressed as total flux/mouse ± SD. **C.** Representative micrographs show two different mammary glands treated i.duc with ^225^Ac-trastuzumab (40 nCi/teat) (a, b). No tumors were visible in these mammary glands under IVIS spectrum imaging examination and by H and E staining. (c) The left 4th and 5th mammary glands were examined by H and E staining. A tumor focus (circle) was identified in the distal area of the left 4th mammary gland macroscopically (d) Microscopic examination revealed SUM225-Luc+ cell growth in the lumen. (e) All 9 mammary glands treated with ^225^Ac-trastuzumab (120 nCi/mouse/i.v) showed massive tumor lesions 28 days after intravenous treatment.

For testing the efficacy of radioimmunotherapy, a week after i.duc implantation of SUM225-Luc+ tumor cells, NSG mice were randomly assigned to five groups before treatment. In each mouse, xenografts in three mammary glands were treated with ^225^Ac-trastuzumab (^225^Ac-T, specific activity: 0.1 μCi/μg) (Figure 4A). Ten, 20 or 40 nCi of ^225^Ac-T/40 μl/teat was administered i.duc to groups of mice (n=3, 3, 4), respectively. 120 nCi of ^225^Ac-T/120 μl per mouse was administered i.v to 3 mice for comparison with the i.duc mode of treatment, and 40 μl of PBS/teat was administered i.duc to 4 teats of 3 mice as vehicle controls. At 28 days after treatment, all i.duc ^225^Ac-T treated groups showed inhibition of tumor growth compared to i.v. ^225^Ac-T and i.duc PBS groups (p<0.0001) (Figure 4B) which carried large tumor burdens leading to the termination of the experiment. There was no significant difference in tumor burden by total flux measurements (Figure S2), but upon histological examination, we found tumor inhibiting efficacy was dose-dependent, with 0/9 (10 nCi), 4/12 (20 nCi), and 11/12 (40 nCi) tumor free mammary glands in each group (Table 1). These results were confirmed by histopathology of the injected mammary glands (Figures 4C). In Figure 4C, representative micrographs of sections of mammary glands in tumor free mice (panel a, b) shows normal appearing ductal structures. Whole mount of the 4^th^ and 5^th^ mammary gland from the tumor bearing mouse in this group (40 nCi/teat), shows tumor growth in the 4^th^ mammary gland (the circled area), and the H and E stained section confirmed the presence of tumor cells in this lesion (panel c, d). Except for mice that received i.v. ^225^Ac-T, no appreciable weight loss was observed in the other treatment groups (Figure S2).

**Table 1:** Response of SUM225 DCIS outgrowth to intraductally administered ^225^Ac-Trastuzumab conjugate

^225^Ac-Trastuzumab					
Treatment	PBS iduc	10 nCi iduc	20 nCi iduc	40 nCi iduc	120 nCi iv
Dose per mouse (nCi)	0	30	60	120	120
No. of mice	2	3	4	4	3 (2†)
No. of tumors/No. of xenograft treated	8/8	9/9	8/12	1/12	9/9

Each of three to four mammary glands per mouse received 10,000 SUM225-Luc+ cells through the teat. All injected glands developed tumors along the ducts. In ^225^Ac-T treated mice, 3 tumor bearing mammary glands in each mouse received ^225^Ac-Trastuzumab conjugate; 2 mice received PBS i.duc into four tumor bearing glands each.

† In mice receiving the conjugate i.v, 2/3 mice died before the end of the experiment at day 17 and 22 of follow up.

Statistical analyses presented in Supp. Tables S1-1 to S1-3

Long-term carcinogenesis is of particular concern with alpha-particle emitters and there are a number of historical examples in which early use of alpha-emitters led to secondary cancers [10]. The extent to which these early examples are relevant to current use of alpha-emitters in targeted therapy is under investigation [10]. We investigated long term toxicity of the ^225^Ac-Trastuzumab conjugate in the mammary gland and distant organs. We administrated 40 nCi of ^225^Ac-T/teat (3 teats in each mouse) i.duc once to female parous FVB/N mice (n=13) and monitored the mice monthly. There was no significant change in body weight during the 13 to 15 month observation period. However, tumors were observed in the mammary gland (1/39 mammary glands, 1/13 mice) and lung (4/13 mice), but not in the liver (0/13) and kidney (0/13) (Supplementary Table S2).

## DISCUSSION

In this paper we report the results of testing the therapeutic effects of radioimmunotherapy with ^225^Ac-T delivered through the intraductal route in a HER-2 positive human DCIS xenograft model in immunodeficient female NSG mice. The DCIS xenograft mouse model provided mammary tumors at a noninvasive stage with high tumor take rates and allowed the study of HER-2 targeted intraductal therapy using ^225^Ac-T. Using this model, we also obtained data on the biodistribution, and therapeutic effectiveness of ^225^Ac-T at doses considerably lower than those used previously for systemic treatment.

The biodistribution studies highlighted the potential advantage of intraductal administration in that systemic exposure was substantially reduced relative to systemic administration of ^225^Ac-Trastuzumab, and the retention of trastuzumab remained at high levels even after 5 days. The therapeutic study using alpha-particle emitter conjugated trastuzumab showed that a total of 120 nCi of i.duc ^225^Ac-Trastuzumab, distributed over three mammary glands inhibited tumor growth significantly more effectively than the same dose administered intravenously (Figure 4). Body weight loss during radioimmunotherapy was lower in the mice treated by the i.duc route compared to the i.v route. Thus, the therapeutic i.duc dose of radioimmunotherapy per teat was lower, which would likely result in reduced hematologic and renal toxicity.

In treating early disease like DCIS, long term toxicity of the therapeutic agent used is always of concern. This was borne out in our previous study in mice using Doxil i.duc [12]. Although highly effective in achieving long term cures in Her2-transgenic mouse tumors [13], i.duc Doxil induced mammary tumors in nearly 70% of naïve FVB/N mice, precluding its use through the intraductal route. ^225^Ac was significantly less carcinogenic in the mammary gland (1/39 mammary glands injected; 1/13 mice), but induced lung tumors in 4/13 mice (Supplementary Table S2). Reducing the dose of the isotope may prevent its escape into the circulation, and eliminate local carcinogenic effects.

The i.duc route was used not only to establish the xenografts, but also to treat the xenografts repeatedly [12–14]. Successful repeated cannulation of the same duct with very high efficiency (97%), 6 months following the first cannulation, has been reported in disease free, high-risk women in a chemoprevention setting [18]. Diseased ducts have been accessed successfully by cannulation for ductal lavage or ductoscopy as shown by us and others [14, 19–21]. Success has been reported in 40-95% of the cases with breast disease. As with all new technologies, wider experience with the technique will lead to better success rates.

We studied radioimmunotherapy using this model at 1 week after inoculation. IVIS spectrum imaging at one week after inoculation showed visible signals of luciferase activity in the inoculated mammary gland, and immunohistochemical staining showed that SUM225-Luc+ cells grew as a 1-2 layers on the mouse luminal epithelial cell layer, leaving ductal lumens open (Figure 3A, 3B). In addition, we observed that therapeutic efficacy of the i.duc ^225^Ac-trastuzumab was significantly higher than i.v administration (Figure 4), without attendant toxicity. These observations warrant further investigation. Given the short-range of the alpha-particles, micro-scale dosimetry will be needed to understand how to best translate these initial results to the clinic [22, 23]

In conclusion, the SUM225 xenograft model is useful to study treatment strategies for DCIS. We have provided evidence in this preclinical model for the potential of intraductal radioimmunotherapy using α-emitter particle ^225^Ac-labeled trastuzumab as a novel therapeutic alternative against DCIS of the breast.

## MATERIALS AND METHODS

### Cell lines

Human breast cancer cell lines, SUM-225 (expressing high endogenous HER-2); MDA-MB-231 and MCF-7 (low endogenous HER2) were maintained in culture according to instructions (ATCC). NT2.5, a cell line derived from Her-2/neu (rat) transgenic mouse mammary tumor, served as a murine negative control (trastuzumab does not bind the rat Her2 transgene). The cells were maintained in culture as previously described [9, 10].

### Animal experiments

Eight to 12-month-old multiparous female mice were used in all the experiments, since prior lactation facilitated intraductal injection with ease and precision. Female NOD.Cg-PrkdcscidIl2rgtm1Wjl/SzJ mice were purchased (NSG mice, The Jackson Laboratory, USA) and bred at Johns Hopkins. Parous FVB/N female mice were purchased from NCI (Frederick, MD) and used in the biodistribution and long term toxicity study. All animal experiments were conducted following protocols approved by Animal Care and Use Committee of JHMI.

### Antibodies

Trastuzumab (Herceptin, Genentech), an anti-human HER-2 IgG monoclonal antibody, and Rituximab (Rituxan, IDEC Pharmaceutical Corp.), an anti-human CD20 IgG monoclonal antibody (non-specific antibody) were used.

### Synthesis of ^111^In and ^225^Ac-labeled antibodies

In the biodistribution study, SCN-CHX-A”-DTPA (Macrocyclics) was conjugated to antibody, and subsequently radiolabeled with ^111^In (2-10μCi/μg mAb) as described previously [9, 10]. ^225^Ac was provided by European Commission, Joint Research Centre, Institute for Transuranium Elements in Karlsruhe, Germany. In the therapeutic study, antibody was conjugated to p-SCN-Bn-DOTA (Macrocyclics) and labeled with ^225^Ac by a 2-step method as described previously [9, 15]. Radiochemical yield of ^225^Ac-DOTA-Trastuzumab was 8.0-14.9% and the purity of ^225^Ac-DOTA-Trastuzumab was 95.1-98.7%; the specific activity of the labeled antibody was 1 μCi/μg mAb.

### Biodistribution of trastuzumab

To examine the difference in pharmacokinetics of trastuzumab injected through two different routes, the antibody was radiolabeled with ^111^In and administered to multiparous female FVB/N wild-type mice (3 mice per group) bearing no tumors by i.v. or i.duc injection. At 2, 24 and 120 hours after injection, mice were sacrificed and major organs and blood were collected. Each organ was measured by a gamma counter (LKB Wallac, Perkin-Elmer). Results were corrected for physical decay and presented as percentage of injected dose per gram (%ID/g). The blood activity concentration profiles of the two different injection routes were compared by using numerical (trapezoidal) integration to obtain the area under the blood time-activity curve (AUC).

### Intraductal transplantation method

Cells were detached from culture plates with 0.25% Trypsin/EDTA and resuspended as single cells in complete growth media and counted. The cell suspension (Matrigel: media =1:1) was placed on ice. A 50 μl Hamilton syringe with a 33-gauge Hamilton metal hub needle (7747-01, N733, point style 3, length 0.375 inches) chilled on ice was used to deliver the cells. The mice were anesthetized under an inhalation anesthesia (isoflurane, -2.0% and oxygen, 1.5L/min). Ten microliters of cell suspension was injected with visualization of the teat under a dissecting microscope (see Supplementary data, video showing methodology of i.duc injection into mouse teats).

### Establishment of luciferase/GFP transfected cells

To enable visualization of the outgrowths, Luciferase and Green Fluorescent Protein (GFP) were introduced into SUM225 cells by retroviral infection. KMRV+Luciferase+GFP plasmid and Ampho-plasmid diluted in OPTi-MEM I were co-transfected into 293T cells with Lipofectamine TM 2000 (Invitrogen), following the manufacturers protocol. Luciferase-transfected (SUM225-Luc+) cells were sorted for GFP using FACS Diva Version 6.1.3. To visualize mouse intraductal DCIS-like xenografts, Bioluminescent imaging using IVIS spectrum (Caliper, Life Sciences, software Living Imaging version 4.2) was conducted.

### Mouse intraductal DCIS-like xenograft model

Ten thousand SUM225-Luc+ cells were transplanted by i.duc injection through the teat into each of 4 mammary glands of three 8-12 month old female NSG mice. Time course of tumor development was determined by staining formalin fixed, paraffin embedded mammary tissue with hematoxylin and eosin (H&E), HER-2 (primary Ab, Cell Signaling Technology, #2242, USA, 1:100 dilutions), red carmine for whole mounts, and IVIS spectrum imaging (IVIS). Histology was performed as described previously [14].

### IVIS spectrum imaging

D-Luciferin, potassium Salt (Gold Biotechnology, LUCK) was dissolved in PBS without Mg^2^+ and Ca^2^+ at a concentration of 15 mg/ml. Mice received 10 μl of filtered D-Luciferin solution per gram body weight intraperitoneally (i.p) 10 minutes before imaging, and mice were then anesthetized. Two to five mice per one image acquisition were examined and all image acquisitions were performed at 30 seconds exposure (Xenogen IVIS Imaging System 100; data analysis using Caliper, Life Sciences, software Living Imaging version 4.2). To compare each group, regions of interest (ROI) were manually selected in squares, including whole body on the image of radiance. Total flux (photons/second) was measured corresponding to each ROI. All total fluxes were used for statistical analysis.

### Specific cell kill *in vitro* by ^225^Ac-trastuzumab

Specific cell kill *in vitro* was determined by colony formation assays. SUM225 cells were seeded into 6-well plates at 0.4 to 5 × 10^3^ cells per well, then treated with serially diluted ^225^Ac-trastuzumab or ^225^Ac-Rituximab (10-30 nCi/ml). After incubation for one hour, SUM225 cells were trypsinized and replated on cell culture Petri dishes for colony growth. Cells were also treated with 1μg/ml cold-trastuzumab for one hour. Survival fraction was calculated using the CFU assay [16].

### Intraductal radioimmunotherapy

40 μl of drug solution was injected into each of three inguinal mammary glands of each mouse as described previously [12–14]. Therapeutic experiments were performed to compare relative efficacy of i.duc compared to the i.v. route of administration.

### Statistical analysis

Quantitative data were expressed as mean ± standard deviation (SD) or percentage when appropriate. Survival distributions were described using Kaplan-Meier method. Analyses of therapeutic efficacy involving the repeated measures data were performed using a hierarchical mixed effects model, where correlations among observations on the same animal were taken into account by assuming an exchangeable covariance structure. Treatment effects were assessed at different time points as total flux over time appeared to differ by treatment. Adjusted P values using Tukey's procedure for multiple testing corrections were provided in addition to the exploratory unadjusted ones. Except for the blood AUC comparison, all statistical tests were two-sided. An unpaired, one tailed t-test was used to assess the significance of the difference in blood AUCs. We chose a one-tailed test because we expect that the AUC following i.duc administration will be lower than after IV administration. P<0.05 was considered statistically significant. The analyses were carried out using GraphPad Prism (v5.0, GraphPad Software, San Diego, CA), SAS software (v9.2, SAS Institute, Cary, NC) or Excel (Microsoft, Inc, Redmund WA).

## SUPPLEMENTARY MATERIALS AND METHODS FIGURES AND TABLES




