# Supplementary material for: Effective treatment of ductal carcinoma in situ with a HER-2-targeted alpha-particle emitting radionuclide in a preclinical model of human breast cancer
Source: Oncotarget. 2016 Apr 23;7(22):33306–15. doi: 10.18632/oncotarget.8949 (PMC5078096; doi:10.18632/oncotarget.8949)
Supplement: Supplementary file 1 [file oncotarget-07-33306-s001.pdf]

SUPPLEMENTARY MATERIALS AND METHODS

Mouse intraductal injection and intraductal therapeutic procedures

All the experiments were conducted on parous mice since prior lactation facilitates intraductal injection. All animal experiments were conducted by following protocols approved by Animal Care and Use Committee of Johns Hopkins University School of Medicine. Hair around each teat is moistened by a forceps with a bit of PCR oil to expose each teat of the mammary gland. Each orifice of the teat is moistened and dilated gently by a top of the fine forceps, the keratin plug if present is teased out, and then a 33G needle is inserted through the teat. Ten microliters of cell suspension is injected under the microscope with appropriate magnification. Little or no leakage from the teat is achieved by slow injection, slow release of the needle from the teat and by holding the tip of the teat with the forceps after release for a few seconds. Avoid cutting the teat prior to cannulation, since this will allow repeat intraductal injections into the same teat, as described previously [1–2].

A video presentation of intraductal injection through the teat of mouse mammary gland is enclosed. The movie shows essential steps of the intraductal injection procedure, as described above. (See Supplementary Video 1).

Statistical analyses

Statistical analyses were carried out using SAS software (version 9.2). All tests were two-sided and considered statistically significant at $P<0.05$. Values for total flux are expressed as $x10^7$.

A hierarchical mixed effects model was used to analyze the repeated measures data, where correlations among observations on the same animal were taken into account by assuming an exchangeable covariance structure. Baseline (Day 0) total flux were considered similar across groups ($P=0.721$). Treatment effects were assessed at different time points as total flux over time appeared to differ by treatment. Adjusted P values using Tukey's procedure for multiple testing corrections were provided in addition to the unadjusted ones.

REFERENCES

1. Murata S, Kominsky SL, Vali M, Zhang Z, Garrett-Mayer E, Korz D, et al. Ductal access for prevention and therapy of mammary tumors. Cancer research 2006;66:638-45.
2. Stearns V, Mori T, Jacobs LK, Khouri NF, Gabrielson E, Yoshida T, Kominsky SL, Huso DL, Jeter S, Powers P, Tarpinian K, Brown RJ, Lange JR, Rudek MA, Zhang Z, Tsangaris TN, Sukumar S. Preclinical and clinical evaluation of intraductally administered agents in early breast cancer. Sci Transl Med. 2011:26;3:106-108.

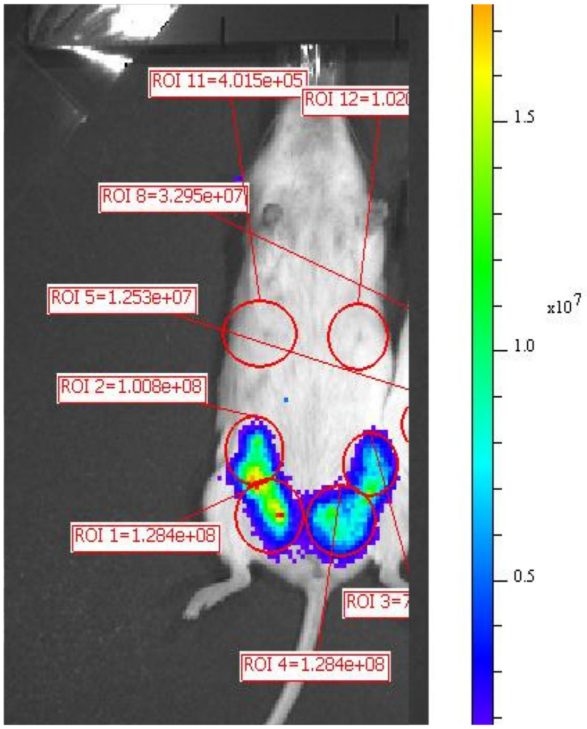

Supplementary Figure S1: Measurement of radiance: Bioluminescent imaging 14 days after inoculation of SUM225-Luc+ cells (10000 cells/teat/10 µl). Exposure time: 30 sec. Mammary glands from mice were subjected to IVIS imaging 14 days after SUM225-Luc+ cell inoculation i.duc. Radiance (photon/sec/cm^2/sr) of the sites injected with Matrigel, and media without cells were measured as background signal at the same time (top circles). Values for total flux are expressed as $x10^7$.

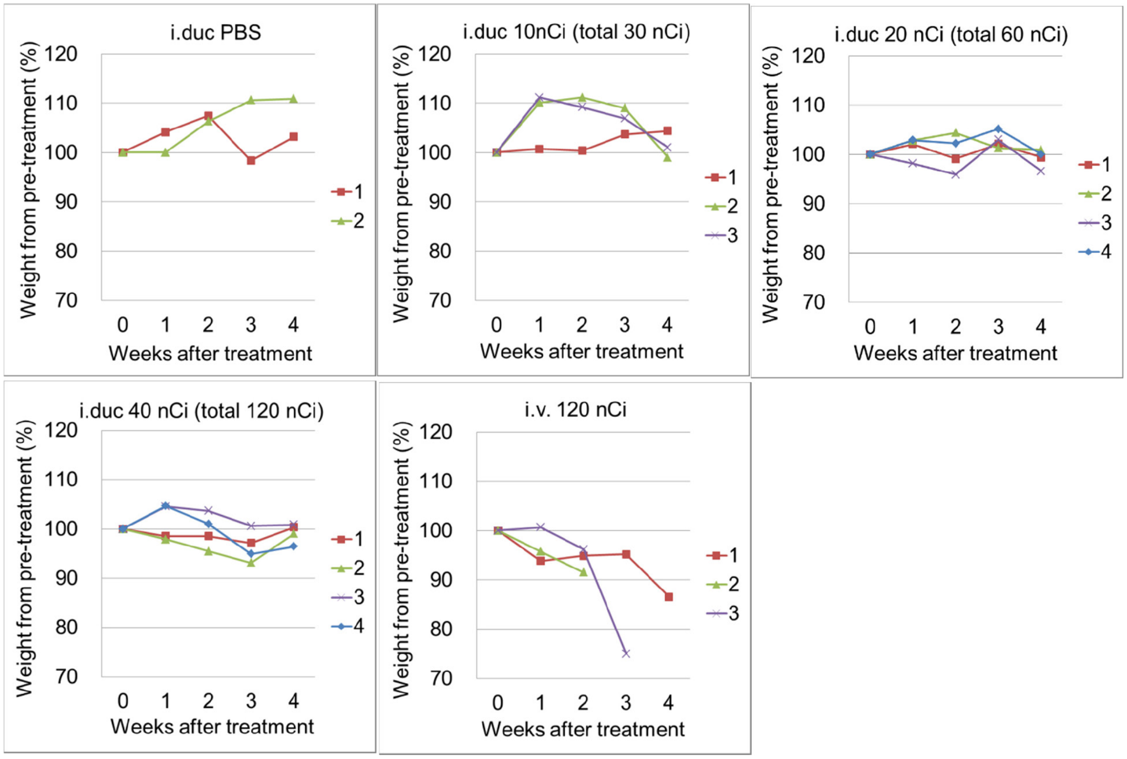

Supplementary Figure S2: Body weight of mice on radioimmunotherapy. Systemic toxicity was evaluated by body weight measurements following intraductal ^{225}Ac-trastuzumab and intravenous ^{225}Ac-trastuzumab in post-breeder female NSG mice. Body weight was measured weekly following tumor implantation to the end of the therapeutic study. Percentage of each initial body weight was plotted each mouse in 5 different groups.

Supplementary Table S1-1: Therapeutic effects of ^{225}Ac-T conjugate on SUM225 DCIS xenografts

Group	Time (day)	N	Mean	Std Dev	Median	Minimum	Maximum
1	0	4	4.2	2.6	3.5	1.9	7.8
	10	4	3.6	2.0	3.2	1.8	6.4
	17	4	2.0	1.5	1.3	1.0	4.2
	28	4	6.0	9.3	1.6	1.0	20.0
2	0	4	3.9	1.7	4.6	1.5	5.1
	10	4	11.7	7.3	11.4	5.0	19.2
	17	4	19.7	7.6	20.9	9.5	27.5
	28	4	77.9	61.1	83.7	14.0	130.3
3	0	3	3.3	0.4	3.3	2.9	3.7
	10	3	24.0	14.3	18.0	13.6	40.3
	17	3	11.9	3.5	13.9	7.8	14.0
	28	3	97.9	42.2	109.6	51.1	132.9
4*	0	3	3.9	1.4	3.6	2.6	5.4
	10	3	69.8	23.4	64.7	49.4	95.4
	17	2	311.9	192.8	311.9	175.6	448.2
5	0	2	2.1	0.8	2.1	1.6	2.7
	10	2	63.7	58.5	63.7	22.4	105.1
	17	2	257.3	116.5	257.3	174.9	339.7
	28	2	1229.6	477.2	1229.6	892.1	1567.0

* In Group 4, only 1 mouse survived until Day 28 and thus no data is shown for d28.

Legend:

Treatment was administered one time at 1 week after inoculation.

Group 1: i.duc 40nCi of ^{225}Ac-trastuzumab/teat (n=4 mice, 3 xenografts/mouse)

Group 2: i.duc 20nCi of ^{225}Ac-trastuzumab/teat (n=4 mice, 3 xenografts/mouse)

Group 3: i.duc 10nCi of ^{225}Ac-trastuzumab/teat (n=3 mice, 3 xenografts/mouse)

Group 4: i.v. 120nCi of ^{225}Ac-trastuzumab/mouse (n=3 mice, 3 xenografts/mouse)

Group 5: i.duc 50 ul of PBS/teat (n=2 mice, 4 xenografts/mouse)

IVIS Spectrum was performed at day0, day10, day17, day28 after treatment.

One ROI was placed to cover the whole body of the mouse and total flux was measured for each mouse.

Supplementary Table S1-2: Model based estimation - least square means for each group at a given time point

Group	Time	Mean total flux	Std Err	Lower limit of 95% CI of mean	Upper limit of 95% CI of mean
1	10	3.7640	41.2853	-86.1856	93.7135
2	10	18.6195	41.2853	-71.3301	108.57
3	10	22.5658	47.6722	-81.2989	126.43
4	10	329.90	53.8921	214.32	445.48
5	10	225.41	58.3862	98.2045	352.62
1	17	4.1286	40.1351	-84.5270	92.7842
2	17	36.6954	40.1351	-51.9602	125.35
3	17	44.3690	46.3440	-58.0017	146.74
4	17	757.06	73.4880	603.28	910.85
5	17	529.25	56.7595	403.87	654.62
1	28	4.7017	82.2298	-160.38	169.78
2	28	65.1004	82.2298	-99.9828	230.18
3	28	78.6310	94.9508	-111.99	269.25
4	28	1428.32	145.20	1135.80	1720.84
5	28	1006.70	116.29	773.24	1240.16

Supplementary Table S1-3: Model based estimation – difference in means between groups at a given time point

The last 3 columns incorporate multiple testing corrections using Tukey's procedure

Group X	Group Y	Time	Estimated mean difference*	Unadjusted P value	Lower limit of 95% CI of mean difference	Upper limit of 95% CI of mean difference	Adjusted p value	Adjusted lower limit of 95% CI of mean difference	Adjusted upper limit of 95% CI of mean difference
1	2	10	-14.8555	0.8035	-142.06	112.35	0.9990	-179.97	150.25
1	3	10	-18.8019	0.7707	-156.20	118.60	0.9982	-197.14	159.54
1	4	10	-326.14	0.0003	-472.57	-179.70	0.0001	-518.12	-134.15
1	5	10	-221.65	0.0092	-377.45	-65.8512	0.0251	-423.87	-19.4308
2	3	10	-3.9464	0.9511	-141.35	133.45	1.0000	-182.29	174.39
2	4	10	-311.28	0.0005	-457.71	-164.85	0.0003	-503.26	-119.30
2	5	10	-206.79	0.0135	-362.59	-50.9957	0.0427	-409.01	-4.5753
3	4	10	-307.33	0.0009	-462.70	-151.97	0.0008	-510.80	-103.86
3	5	10	-202.85	0.0196	-367.07	-38.6219	0.0693	-416.00	10.3096
4	5	10	104.49	0.2115	-67.3581	276.33	0.6832	-120.21	329.18
1	2	17	-32.5668	0.5780	-157.94	92.8112	0.9783	-193.08	127.94
1	3	17	-40.2403	0.5255	-175.66	95.1834	0.9647	-213.61	133.13
1	4	17	-752.94	<.0001	-930.11	-575.76	<.0001	-989.73	-516.15
1	5	17	-525.12	<.0001	-678.67	-371.56	<.0001	-721.70	-328.53
2	3	17	-7.6736	0.9027	-143.10	127.75	0.9999	-181.04	165.70
2	4	17	-720.37	<.0001	-897.55	-543.19	<.0001	-957.16	-483.58
2	5	17	-492.55	<.0001	-646.11	-338.99	<.0001	-689.13	-295.97
3	4	17	-712.70	<.0001	-897.04	-528.35	<.0001	-958.38	-467.01
3	5	17	-484.88	<.0001	-646.74	-323.01	<.0001	-692.09	-277.66
4	5	17	227.82	0.0269	29.8794	425.76	0.1178	-34.7671	490.40
1	2	28	-60.3987	0.6057	-293.86	173.06	0.9850	-389.26	268.46
1	3	28	-73.9293	0.5587	-326.10	178.24	0.9762	-429.14	281.28
1	4	28	-1423.62	<.0001	-1759.27	-1087.98	<.0001	-1895.51	-951.73
1	5	28	-1002.00	<.0001	-1287.93	-716.06	<.0001	-1404.76	-599.23
2	3	28	-13.5306	0.9146	-265.70	238.64	1.0000	-368.74	341.68
2	4	28	-1363.22	<.0001	-1698.87	-1027.58	<.0001	-1835.11	-891.33
2	5	28	-941.60	<.0001	-1227.53	-655.67	<.0001	-1344.36	-538.83
3	4	28	-1349.69	<.0001	-1698.56	-1000.83	<.0001	-1840.31	-859.07
3	5	28	-928.07	<.0001	-1229.47	-626.67	<.0001	-1352.62	-503.51
4	5	28	421.62	0.0279	47.7024	795.55	0.1727	-104.45	947.70

Supplementary Table S2: Tumor incidence in female FVB/N mice injected i.duc with ^{225}Ac-T conjugate

Tumor incidence	Mammary gland	Lung	Kidney	Liver
No. of mice (n=13)	1/13	4/13	0/13	0/13
No. of treated mammary glands	1/39	-	-	-
No. of untreated mammary glands	0/91	-	-	-

Long term toxicity test was conducted in 16 week old, multiparous, female FVB/N mice. Three teats per mouse received i.duc injection of 40 nCi ^{225}AC-T conjugate per teat. Mice were observed for 12-15 months for tumor development.
